# Maternal and perinatal outcomes in mixed antenatal care modality implementing telemedicine in the southwestern region of Colombia during the COVID-19 pandemic

**DOI:** 10.1186/s12913-023-09255-4

**Published:** 2023-03-16

**Authors:** María Fernanda Escobar, Juan Carlos Gallego, María Paula Echavarria, Paula Fernandez, Leandro Posada, Shirley Salazar, Isabella Gutierrez, Juliana Alarcon

**Affiliations:** 1 High Complexity Obstetric Unit, Department of Obstetrics and Gynecology, Fundación Valle del Lili, Cra 98 Nro.18-49, 7600.2 Cali, Colombia; 2 Department of Obstetrics and Gynecology, School of Medicine, Universidad Icesi, Cali, Colombia; 3 Department of Telemedicine, Fundación Valle del Lili, Cali, Colombia; 4 Centro de Investigaciones Clínicas, Fundación Valle del Lili, Cali, Colombia

**Keywords:** Telemedicine, Maternal–Fetal medicine, Antenatal care, eHealth

## Abstract

**Introduction:**

Contingency measures due to the COVID-19 pandemic limited access to routine prenatal care for pregnant women, increasing the risk of pregnancy complications due to poor prenatal follow-up, especially in those patients at high obstetric risk. This prompted the implementation and adaptation of telemedicine.

**Objective:**

We aim to evaluate the maternal and perinatal outcomes of patients who received prenatal care in-person and by telemedicine.

**Methods:**

We conducted a retrospective observational cohort study of pregnant women who received exclusive in-person and alternate (telemedicine and in-person) care from March to December 20,202, determining each group's maternal and neonatal outcomes.

**Results:**

A total of 1078 patients were included, 156 in the mixed group and 922 in the in-person group. The patients in the mixed group had a higher number of prenatal controls (8 (6–9) vs 6 (4–8) *p* < 0.001), with an earlier gestational age at onset (7.1 (6–8.5) vs 9.3 (6.6–20.3), *p* < 0.001), however, they required a longer hospital stay (26 (16,67%) vs 86 (9,33%), *p* = 0.002) compared to those attended in-person; there were no significant differences in the development of obstetric emergencies, maternal death or neonatal complications.

**Discussion:**

Incorporating telemedicine mixed with in-person care could be considered as an alternative for antenatal follow-up of pregnant women in low- and middle-income countries with barriers to timely and quality health care access.

## Introduction

Maternal and perinatal health are fundamental pillars of a country's public health, being one of the Sustainable Development Goals, so efforts focused on their improvement must always be present [1]. In 2017, the global maternal mortality ratio (MMR) was 211 per 100,000 live births (LB), with almost 99% of deaths registered in low- and middle-income countries (LMIC) [2]; for this same year, Colombia recorded an MMR of 50.7 per 100,000 LB [3]. In 2021, two years after the outbreak of the novel coronavirus pandemic, the MMR in Colombia was 78.3 deaths per 100,000 LB, and nine territorial entities registered a higher than 100 per 100,000 LB [4]. Since the COVID-19 pandemic, a global increase in maternal mortality, near-miss mortality, perinatal mortality, and neonatal morbidity is observed, being more significant in LMICs [5, 6].

Therefore, in confinement settings with a high social cost and economic detriment, it is urgent to prioritize equitable access to high-quality maternal care [5, 7]. Prenatal care has been associated with a decrease in maternal and perinatal mortality and complications related to pregnancy such as hypertensive disorders, intrauterine growth restriction, and preterm delivery [8–11]. The measures taken to address the health emergency by COVID-19 such as the adaptation of the infrastructure and the redistribution of health personnel to support and optimize emergency rooms, intensive care units, and hospitalization services, as well as the relocation in telework of those health workers with high-risk comorbidities, led to a lack of coverage and a reduction of quality prenatal care increasing pregnancy complications [11, 12]. To confront the problem, different organizations encouraged the use of telehealth [11, 13, 14], the use of these new tools in the setting of pregnant women has focused on the management of complications such as gestational diabetes, hypertensive disorders, and obesity [15–19]. Evidence on synchronous telemedicine in prenatal care is limited; however, it has shown similar results compared with exclusively in-person prenatal care [20–23].

Fundación Valle del Lili is a quaternary level university hospital located in Cali, which receives highly complex patients referred from the southwestern areas of Colombia. At the beginning of the mandatory confinement in Colombia, the hospital implemented an outpatient telehealth system through videoconference tools [24], which allowed the follow-up of patients from 65 medical specialties, including obstetric patients. The main objective of this research was to compare maternal and perinatal outcomes between pregnant patients who received their prenatal care in an alternate check-up (telemedicine and In-person) and those who received it exclusively in-person.

## Materials and methods

### Design

We conducted a retrospective observational cohort study of obstetrics patients attended for prenatal care from March 1 to December 31, 2020, at the Fundación Valle del Lili, divided into two groups:

- ­ Mixed or Telemedicine cohort: those patients who received prenatal check-ups alternating between telemedicine and in-person, with compliance with at least one consultation via telemedicine.
- ­ In-person cohort: patients who received exclusively in-person prenatal check-ups.

Patients were free to choose if they wanted in-person or telemedicine care according to preference. Patients who needed vital signs or a physical examination to define the treatment plan were directed to receive an in-person check-up. However, no specific medical condition was considered a contraindication for telemedicine follow-up. Records with incomplete prenatal care data and patients who performed check-ups at other institutions were excluded.

The institutional biomedical research ethics committee approved the protocol of this study, informed consent was not required for the study as it was classified as risk-free according to national resolution (No. 008430 of 1993, article 11, numeral A) of the Ministry of Health and Social Protection of Colombia.

### Data collection and variables

We used the ICD-10 codes to identify the pregnant women who received prenatal follow-up at the institution in the period from March 1 to December 31, 2020, the statistics and information management department in conjunction with the telemedicine program identified the patients who received care by telemedicine and those who received exclusively in-person care. We then collected data from the institutional electronic medical record, which were registered in the BdClinic database software. Table 1 shows the variables that were considered to evaluate maternal and neonatal outcomes.

**Table 1:** Maternal and neonatal variables considered to determine outcomes

Maternal Variables	Neonatal Variables
­ Delivery route or termination of pregnancy ­ Early hospital discharge^a^ ­ Admission to the high complexity obstetrics unit (HCOU)^b^ ­ Access to the intensive care unit ­ Development of obstetric emergency ­ Maternal death	­ Newborn birth weight ­ Need for hospitalization after childbirth ­ Admission to the neonatal intensive care unit ­ Neonatal death

^a^ Discharge within the first 48 h after delivery

^b^ High dependency unit for the management of pregnancies classified as high obstetric risk

### Implementation of telemedicine in antenatal care

In response to the health emergency caused by the COVID-19 pandemic, the Fundación Valle del Lili implemented and adapted telemedicine strategies by developing the " Siempre" (Always) program as an alternative for outpatient care, avoiding the spread and exposure to the virus.

The "Siempre" program uses the Microsoft Teams platform, which establishes real-time video calls between the obstetrician-gynecologist and the pregnant women. The clinical practice guidelines of the Colombian Ministry of Health and Social Protection recommend at least ten prenatal check-ups for nulliparous women and seven for multiparous women [25]; however, this guide does not contemplate telemedicine interventions, so the recommendations of the American Society of Gynecology and Obstetrics—Maternal–Fetal Medicine delivered in the guide "MFM guidance for COVID-19" (Table 2) were considered [26]. Women could have more or fewer evaluations than those established, depending on the risk classification according to the validated scale of Herrera and Hurtado [27] and on clinical needs; those patients with high obstetric risk check-ups were programmed monthly until 37 weeks of gestational age, then check-ups were every 15 days until the delivery.

**Table 2:** Telemedicine care program integrated into prenatal care in Fundación Valle del Lili

Gestational age	Obstetrics and gynecology follow-up		Ultrasounds and Labs
	**Via In-Person**	**Via Telemedicine**	
**< 11 weeks**		X	
**11 – 13 weeks**	X		X
**18 – 24 weeks**	X		X
**28 weeks**	X		
**32 weeks**		X	X
**36 weeks**	X		X (As required)
**38 weeks to Birth**	X		
**Postpartum**		X	

Throughout the medical attention, the obstetrician-gynecologist updates the patient's current condition, asks about the presence of symptoms or warning signs, the need for emergency consultations or hospitalizations since the last check-up, and analyzes the last examinations performed. The information is recorded in the institutional electronic medical record system, through which the request for procedures, paraclinical or imaging tests, referral for evaluation by other necessary specialties, and formulation of medications are made. The modality and date of the next appointment are decided with the patient at the end of the video call, and the documents generated are sent in PDF format to each patient's e-mail address.

### Statistical analysis

The research conducted by Yvonne Butler et. al [22], estimated an incidence of cesarean delivery of 15% in pregnant women who were attended in-person and 13% in those attended via telemedicine; with these data, a sample size of 9,448 (4,724 for each group) was calculated for a confidence level of 95% and a power of 80%. However, the institutional department of statistics and information management indicated that during the defined period a total of 1808 prenatal care were performed, being 190 via telemedicine, which offers a power of 81% with a 95% confidence level to identify 15% of cesarean deliveries in in-person consultations and 23% via telemedicine, with an Unexposed/Exposed ratio of 25%, requiring 940 patients attended exclusively in person.

A total of 1,618 in-person visits were carried out in the determined period, so a simple random sampling was performed by enumerating each patient, and then the 922 patients were randomly obtained using the Random number generator Comprehensive Version on the calculator.net website.

The Shapiro–Wilk test was used to evaluate the normality distribution of the numerical variables, taking a *p*-value < 0.05 as a significance value; thus, medians and their respective interquartile ranges were used to describe these variables. The qualitative variables were summarized through percentages and presented in frequency tables. The Mann–Whitney-Wilcoxon statistical tests were used to evaluate the difference in the sociodemographic or clinical numerical variables between cohorts, while the differences in the qualitative variables were evaluated using the Chi2 test.

The Chi2 test will be used to determine the differences in each maternal and perinatal outcome incidence. In case of being a dichotomous outcome and the expected value is less than 5 in any category, Fisher's test will be used. Statistically significant differences will be considered if the *p*-value < 0.05.

## Results

**Fig. 1:**
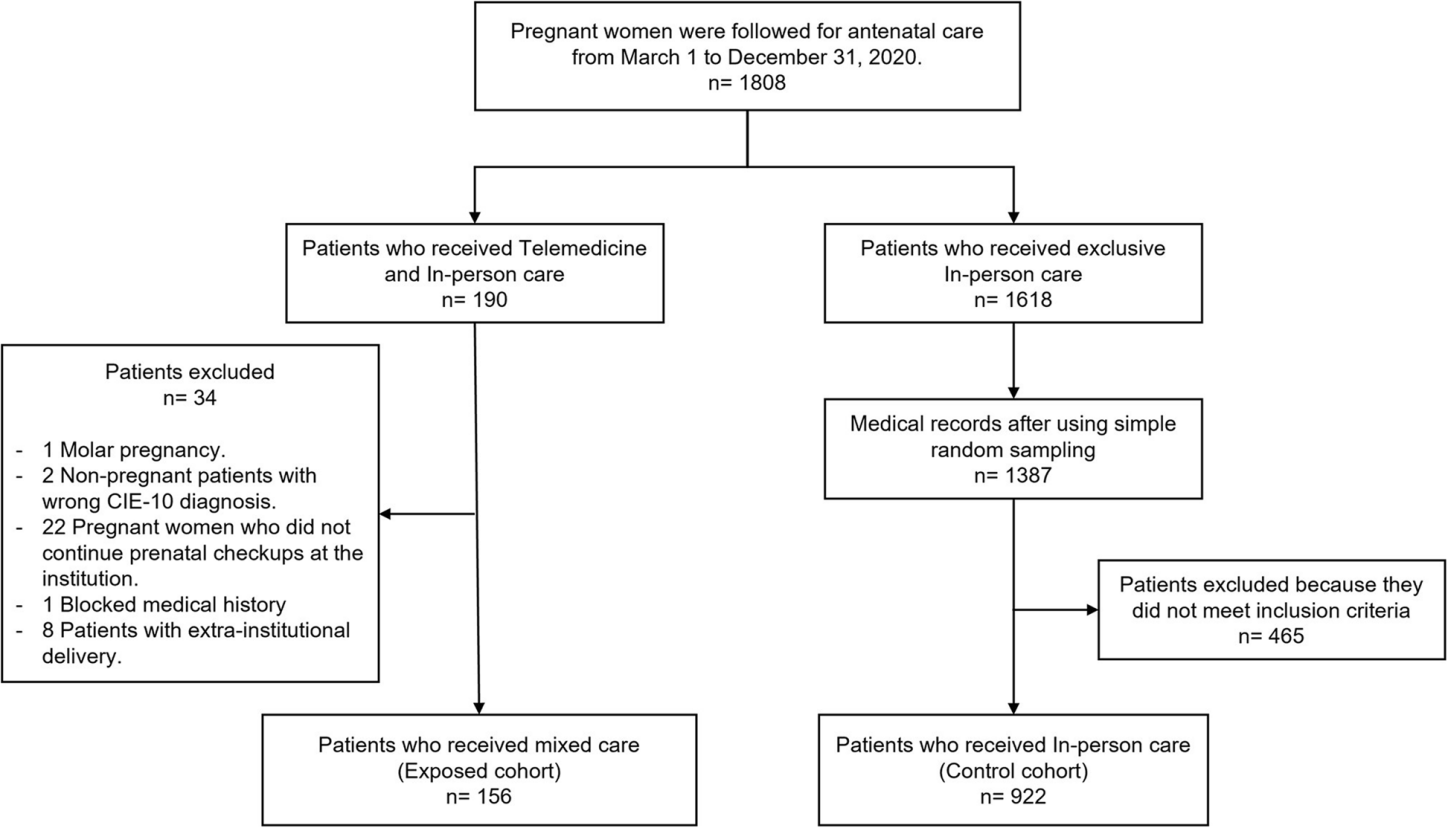
Distribution of patients with antenatal check-ups by telemedicine versus exclusively in-person modality of attention

A total of 1808 patients received antenatal care check-ups (190 via telemedicine, and 1618 in-person) from March 1 to December 31, 2020. After using simple random sampling for the in-person group and applying inclusion and exclusion criteria we obtained 156 (14.5%) patients attended by a Mixed modality, being the exposed cohort; and 922 (85.5%) patients in-person modality, being the control cohort (Fig. 1).

The median age was 31 (IQR 28–35), and most of the participants lived in urban areas (95,27%); in the mixed group 63,46% had a bachelor’s degree being higher than the in-face group (48,26%, *p* = 0.041) the other sociodemographic characteristics were no statistically significant differences between both cohorts. Women in the mixed group started their routine antenatal care earlier with a median gestational age at onset of 7.1 weeks (IQR 6—8.5) versus 9.3 weeks (IQR 6.6—20.3) in the in-person group (*p* < 0.001); they also presented a greater number of antenatal appointment received than the in-person group (8 [IQR 6–9] vs. 6 [IQR 4–8], *p* < 0.001) (Table 3).

**Table 3:** Sociodemographic and clinical characteristics of the pregnant women in the mixed group vs. In-person group

Variables	Total (*n* = 1078)	Mixed antenatal control (*n* = 156)	In-person antenatal control (*n* = 922)	*P* value
Age, years, median (IQR)	31 (28—35)	31 (28—35)	31 (28—35)	0.7608
Area of residence, n (%)				
Urban area	1027 (95,27)	151 (96,79)	876 (95,01)	0.555
Rural area	28 (2,60)	3 (1,92)	25 (2,71)	
No data	23 (2,13)	2 (1,23)	21 (2,28)	
Occupation, n (%)				
Unemployed	51 (4,73)	9 (5,77)	42 (4,56)	0.263
Employed	659 (61,13)	107 (68,59)	552 (59,87)	
Independent	100 (9,28)	10 (6,41)	90 (9,76)	
Housewife	119 (11,04)	14 (8,97)	105 (11,39)	
No data	149 (13,82)	16 (10,26)	133 (14,43)	
Scholarship, n (%)				
Basic	5 (0,46)	0 (0)	5 (0,54)	0.041
High school	256 (23,75)	28 (17,95)	228 (24,73)	
Bachelor degree	544 (50,46)	99 (63,46)	445 (48,26)	
Master's or doctorate	68 (6,31)	9 (5,77)	59 (6,40)	
No data	205 (19,02)	20 (12,82)	185 (20,07)	
Number of antenatal contacts, median (IQR)	7 (4 – 8)	8 (6—9)	6 (4—8)	< 0,001*
Gestational age at the time of admission to routine antenatal care, weeks (IQR)	8.5 (6.4 – 17.3)	7.1 (6 – 8.5)	9.3 (6.6 – 20.3)	< 0,001*
Gestational age at pregnancy termination ^a^ (weeks), median (IQR)	38.2 (37.3 – 39)	38.2 (37.2 – 39)	38.2 (34.7—39)	0,498
High-risk pregnancy, n (%)	702 (65,12)	108 (69,23)	594 (64,43)	0.244
Emergency admission, n (%)	529 (49,07)	69 (44,23)	460 (49,89)	0.191
Gravidity, median (IQR)	2 (1—2)	2 (1—2)	2 (1—2)	0.189
Twin pregnancy, n (%)	17 (1,58)	2 (1,28)	16 (1,63)	0.749

Abbreviations: *IQR* Interquartile range, *AC* Antenatal control

^*^ *p*-value less than 0.001

We used the Herrera & Hurtado scale to classify the biopsychosocial risk of the pregnant woman, finding an elevated percentage of high-risk pregnancies in the total sample (65,12%), without differences between cohorts (Mixed 108 (69,23%) vs. In-person 594 (64,43%), *p* = 0.244). Likewise, admission to the emergency room for obstetric causes during the gestational period was similar for both cohorts (Mixed 69 (44,23%) vs. In-person 460 (49,89%), *p* = 0.191) (Table 3).

Regarding maternal outcomes, 72,26% of the total patients required cesarean section as a way of delivery, without difference between both groups (Mixed 117 (75%) vs. In-person 662 (71,8%), *p* = 0.424. After the attention of the obstetric event, both groups had a higher percentage of women with an early hospital discharge, but with a higher frequency in the prolongation of the hospitalization time in the mixed group (mixed 26 (16,67%) vs. in-person 86 (9,33%) *p* = 0.002). Additionally, we evaluated the need for admission to the High complexity obstetric unit for the surveillance and care of childbirth, evidencing a higher frequency of admission in the in-person group (mixed 39 (25%) vs. in-person 332 (36,01%), *p* = 0.005). None of the other maternal outcomes evaluated reached a statistically significant difference between cohorts (Table 4).

**Table 4:** Maternal outcomes of women treated via telemedicine vs. In-person

Variable	Total (*n* = 1078)	Mixed antenatal control (*n* = 156)	In-person antenatal control (*n* = 922)	*P* value
**Final state of pregnancy** ^**a**^**, n (%)**				
Vaginal delivery	265 (24,58)	32 (20,51)	233 (25,27)	0.424
Cesarean section	779 (72,26)	117 (75)	662 (71,8)	
Abortion	34 (3,15)	7 (4,49)	27 (2,92)	
**Hospital discharge, n (%)**				
Not hospitalized	29 (2,69)	8 (5,13)	21 (2,28)	0.002
Late hospital discharge ^c^	112 (10,39)	26 (16,67)	86 (9,33)	
Early hospital discharged ^b^	935 (86,73)	122 (78,21)	813 (88,18)	
**Admission to HCOU, n (%)**	371 (34,42)	39 (25)	332 (36,01)	0.005
**Admission to ICU, n (%)**	38 (3,53)	6 (3,85)	32 (3,47)	0.838
**Obstetric emergency, n (%)**	405 (37,57)	63 (40,38)	342 (37,09)	0.845
**Maternal mortality, n (%)**	0	0	0	N/A

Abbreviations: *HCOU* High complexity obstetric unit, *ICU* Intensive care unit. N/A: Not apply

^a^ This variable included: Abortion, voluntary interruption of pregnancy, vaginal delivery, and cesarean section

^b^ Late hospital discharge: before 48 h after delivery

^c^ Early hospital discharge: within the first 48 h after delivery

Finally, no statistically significant differences were identified between groups in the evaluated neonatal outcomes. There was a greater number of newborns with an adequate birth weight (2500—3999 g: mixed 127 (81,41%) vs. in-person 792 (85,9%), *p* = 0.171), most of the newborns did not require hospitalization (Mixed 104 (66,67%) vs. in-person 621 (67,35%), *p* = 0.435), a lower percentage required admission to the ICU / Neonatal (mixed 17 (10,90%) vs. in-person 122 (13,63%), *p* = 0.453) and only 29 deaths occurred (mixed 7 (4,49%) vs. in-person 22 (2,38%), *p* = 0.088) (Table 5).

**Table 5:** Neonatal outcomes of women attended mixed vs. In-person modalities

Variable	Total (*n* = 1078)	Mixed antenatal control (*n* = 156)	In-person antenatal control (*n* = 922)	*P* value
**Newborn weight, n (%)**				
< 500 gr	6 (0,56)	2 (1,28)	4 (0,43)	0.171
500—999 gr	4 (0,37)	2 (1,28)	2 (0,22)	
1000—1499 gr	3 (0,28)	0 (0)	3 (0,33)	
1500—2499 gr	86 (7,98)	15 (9,62)	71 (7,70)	
2500—3999 gr	919 (85,25)	127 (81,41)	792 (85,90)	
≥ 4000 gr	40 (3,71)	6 (3,85)	34 (3,69)	
**Hospital discharge, n (%)**				
Not hospitalized	725 (67,25)	104 (66,67)	621 (67,35)	0.435
Late hospital discharge ^a^	160 (14,84)	19 (12,18)	141 (15,29)	
Early hospital discharged ^b^	134 (12,43)	23 (14,74)	111 (12,04)	
No data	59 (5,47)	10 (6,41)	49 (5,31)	
**Admission to Neonatal ICU, n (%)**	139 (12,89)	17 (10,90)	122 (13,63)	0.453
**Perinatal mortality, n (%)**				
No perinatal mortality	1024 (94,99)	146 (93,56)	878 (95,23)	0.088
Before childbirth	23 (2,13)	6 (3,85)	17 (1,84)	
Intrapartum	2 (0,19)	1 (0,64)	1 (0,11)	
Postpartum	4 (0,37)	0	4 (0,43)	

Abbreviations: *ICU* Intensive care unit

^a^ Late hospital discharge: before 48 h after delivery

^b^ Early hospital discharge: within the first 48 h after delivery

## Discussion

The maternal and perinatal outcomes of women who received attention in mixed modality prenatal care (in-person plus telemedicine) were similar to those recorded in women with exclusively in-person care. These results indicate no adverse impact with the incorporation of mixed modality telemedicine in antenatal follow-up compared with exclusively in-person visits. Inclusive, telemedicine allowed earlier admission to the program and a more significant number of evaluations related to greater maternal satisfaction and decreased adverse outcomes, such as perinatal mortality [28].

For pregnant women, the COVID-19 pandemic and government restrictions imposed to prevent the spread of the disease led to delayed entry to prenatal care, less care, and increased related complications [12, 29]. The disruption of services focused on maternal and newborn health, mainly in low and middle-income countries, leads to a significant increase in the number of maternal and perinatal deaths, and even a reduction in coverage and an increase in waiting times of 10% can contribute to 253,000 perinatal deaths and 12,200 additional maternal deaths [30]. Faced with situations such as the current contingency, seeking strategies to avoid this lack of continuity in services is essential to prevent indirect complications. Because of that, we highlight how an outpatient teleconsultation program allowed an earlier admission to the antenatal program and a higher number of evaluations, managing to face the current health problem. This is how different organizations promoted the integration of telemedicine tools into prenatal control programs, leading to restructure the traditional in-person services programs [30, 31].

Although the pandemic promoted telemedicine, this is not a new practice in prenatal care. Butler Tobah et al. conducted a randomized clinical trial to evaluate the effectiveness of an antenatal program for low-risk women, which reduced the number of in-person care and were replaced by virtual visits and monitoring devices without differences between groups and with had greater satisfaction and less stress related to pregnancy in the mixed group [22]. With the arrival of the pandemic, multiple health centers around the world implemented new care protocols that promoted social distancing using telehealth tools. However, the evidence so far has focused on the description of these programs carried out in developed countries, with little information regarding the clinical outcomes of patients and newborns [32]. Prospective studies are therefore needed to allow a better comparison of prenatal care modalities among different risk groups of pregnant women in low- and middle-income countries.

Even in high-income countries, one of the concerns of incorporating telemedicine in prenatal care programs is the applicability of this modality in high-risk pregnant women. In our case, we have highlighted how most of the women treated fell into this category. Since no specific clinical condition limited the use of any of the care modalities, no difference was found in this condition between both cohorts. Telemedicine tools have proven useful to reduce in-person care when these are not viable in pregnancies with low and high risk with specific conditions such as gestational diabetes, cardiovascular diseases, and fetal and genetic disorders, leading to avoiding unnecessary exposure of women to hospital environments [21, 33]. Published studies found no significant differences in the incidence of intrauterine growth restriction, preeclampsia, gestational diabetes, or neonatal morbidity and mortality [34]. Our results are aligned, having found no differences in the causes of obstetric emergency at the time of birth. The high cesarean rate reported in both groups (61.1% in the telemedicine group and 65.5% in the in-person group) is related, above the national average reported in 2019 (44.5%) [35], possibly due to the high clinical complexity of the women managed in our institution. Likewise, the length of hospital stay for the care of the obstetric event (which was longer in the telemedicine group) may be associated with the way of termination of pregnancy since cesarean delivery has been identified as an independent factor to prolong the stay, with an average follow-up between 2.5—9.3 days [36].

With these results, one of the most significant discussions is the possibility of extending this care modality to the Colombian territory and other LMICs. The coverage of antenatal programs in Colombia continues to be low, with 62.9% of all births with less than seven evaluations, and 4.8% without any attention, according to national reports for 2019 [37]. Additionally, according to the country's Ministry of Information Technologies and Communications, in Colombia, there is a wide gap in fixed internet access between regions, with the access of 25.3 connections for every 100 residents in Bogotá, up to 5 for every 100 inhabitants in the Guaviare or Guainía, departments with the lowest human development index [38]. Telemedicine coverage is more complex in these areas with less internet access, and paradoxically, the highest MMRs are concentrated. Duryea et al. [23] implemented a prenatal care program where synchronous telemedicine was incorporated using only audio and compared perinatal outcomes with those who received conventional management. Consistent with what was found in our research, the telemedicine group had an earlier admission to prenatal care (11 weeks) and a more significant number of evaluations (9.8 vs. 9.4). They found no differences in neonatal mortality, admission to the NICU, hypertensive disorders, postpartum hemorrhage, or type of delivery. They demonstrated how it is possible to expand antenatal services' coverage through different telehealth modalities allowing early admission and increased follow-up during gestation, without increasing adverse clinical outcomes, which could be an alternative to explore in regions with low internet speed.

### Limitations

The retrospective nature of the research leads to a higher risk of biases, which we tried to control by including all pregnant women evaluated with at least one assessment via telemedicine in the exposed cohort and using random sampling to select the control group. Additionally, after applying exclusion criteria, we could not reach the sample size initially proposed, which could limit the identification of statistically significant differences between groups. Therefore, it would be important to carry out prospective studies comparing the modalities of care in low and middle-income countries, making it possible to evaluate the results in relation to temporality.

## Conclusions

The results of this research are encouraging by showing telemedicine as a tool that increases access to healthcare, leads an earlier entry into prenatal control, and allows continuing with the delivery of an effective and quality prenatal control, even if it is only a single check-up; showing itself as an alternative when in-person evaluation is not the first option. Although this program was quickly implemented during the COVID-19 contingency and understanding the limitations of telehealth to perform a physical examination that is essential in pregnant patients, the benefits found may prompt the use of telemedicine to expand coverage of the prenatal control programs in LMIC. The following steps will be to build and validate safe, quality alternating care models based on this evidence, which will accelerate the adoption of technology and reduce the use of technologies for the care of pregnant women.

## Data Availability

The datasets analyzed during the current study and that support the findings of this study are available from Fundación Valle del Lili, but restrictions apply to the availability of these data, due to internal privacy policies. Data are however available from Escobar MF. Upon reasonable requests and with permission of Fundación Valle del Lili.

## References

[1] Kruk ME, Gage AD, Arsenault C (2018). High-quality health systems in the Sustainable Development Goals era: time for a revolution. Lancet Glob Health.

[2] WHO, Unicef, UNFPA, And WBG, D. UNP, World Health Organization. Trends in maternal mortality (2000). to 2017.

[3] Instituto Nacional de Salud. INFORME DE EVENTO MORTALIDAD MATERNA, COLOMBIA, AÑO 2017. Bogotá; 2018 Apr.

[4] Instituto Nacional de Salud de Colombia. Boletín Epidemiológico Semanal Semana epidemiológica 52. 26 de diciembre del 2021 al 1 de enero de 2022. https://www.ins.gov.co/buscador-eventos/BoletinEpidemiologico/2021_Boletin_epidemiologico_semana_52.pdf

[5] Chmielewska B, Barratt I, Townsend R, Kalafat E, van der Meulen J (2021). Effects of the COVID-19 pandemic on maternal and perinatal outcomes: a systematic review and meta-analysis. Lancet Glob Health.

[6] Villar J, Ariff S, Gunier RB, Thiruvengadam R (2021). Maternal and neonatal morbidity and mortality among pregnant women with and without COVID-19 infection: the intercovid multinational cohort study. JAMA Pediatr.

[7] Bonaccorsi G, Pierri F, Cinelli M, Flori A, Galeazzi A (2020). Economic and social consequences of human mobility restrictions under COVID-19. Proc Natl Acad Sci U S A.

[8] Bishai DM, Cohen R, Alfonso YN, Adam T, Kuruvilla S, Schweitzer J (2016). Factors contributing to maternal and child mortality reductions in 146 low- and middle-income countries between 1990 and 2010. PLoS One.

[9] Shiferaw K, Mengiste B, Gobena T, Dheresa M (2021). The effect of antenatal care on perinatal outcomes in Ethiopia: A systematic review and meta-analysis. PLoS One.

[10] Countdown to 2030 Collaboration (2018). Countdown to 2030: tracking progress towards universal coverage for reproductive, maternal, newborn, and child health. Lancet.

[11] Bhutta ZA, Black RE (2013). Global maternal, newborn, and child health–so near and yet so far. N Engl J Med.

[12] Roberton T, Carter ED, Chou VB, Stegmuller AR, Jackson BD, Tam Y (2020). Early estimates of the indirect effects of the COVID-19 pandemic on maternal and child mortality in low-income and middle-income countries: a modelling study. Lancet Glob Heal.

[13] Turrentine M, Ramirez M, Monga M, Gandhi M, Swaim L, Tyer-Viola L (2020). Rapid deployment of a drive-through prenatal care model in response to the coronavirus disease 2019 (COVID-19) pandemic. Obstet Gynecol.

[14] Kasaven LS, Saso S, Barcroft J, Yazbek J, Joash K, Stalder C (2020). Implications for the future of obstetrics and gynaecology following the COVID-19 pandemic: a commentary. BJOG An Int J Obstet Gynaecol.

[15] Van Den Heuvel JFM, Groenhof TK, Veerbeek JHW, Van Solinge WW, Lely AT, Franx A (2018). eHealth as the next-generation perinatal care: An overview of the literature. J Med Internet Res.

[16] Xie W, Dai P, Qin Y, Wu M, Yang B, Yu X (2020). Effectiveness of telemedicine for pregnant women with gestational diabetes mellitus: An updated meta-analysis of 32 randomized controlled trials with trial sequential analysis. BMC Pregnancy Childbirth.

[17] Kalafat E, Benlioglu C, Thilaganathan B, Khalil A (2020). Home blood pressure monitoring in the antenatal and postpartum period: A systematic review meta-analysis. Pregnancy Hypertens.

[18] Khalil A, Perry H, Lanssens D, Gyselaers W (2019). Telemonitoring for hypertensive disease in pregnancy. Expert Rev Med Devices.

[19] Ferrara A, Hedderson MM, Brown SD, Ehrlich SF, Tsai AL, Feng J (2020). A telehealth lifestyle intervention to reduce excess gestational weight gain in pregnant women with overweight or obesity (GLOW): a randomized, parallel-group, controlled trial. Lancet Diabetes Endocrinol.

[20] Greiner AL (2017). Telemedicine applications in obstetrics and gynecology. Clin Obstet Gynecol.

[21] Palmer K, Davies-Tuck M, Tanner M, Rindt A, Papacostas K, Giles M (2021). Widespread implementation of a low-cost telehealth service in the delivery of antenatal care during the COVID-19 pandemic: an interrupted time-series analysis. Lancet.

[22] Butler Tobah YS, LeBlanc A, Branda ME, Inselman JW, Morris MA, Ridgeway JL (2019). Randomized comparison of a reduced-visit prenatal care model enhanced with remote monitoring. Am J Obstet Gynecol.

[23] Duryea EL, Adhikari EH, Ambia A, Spong C, McIntire D, Nelson DB (2021). Comparison between in-person and audio-only virtual prenatal visits and perinatal outcomes. JAMA Netw Open.

[24] Escobar MF, Henao JF, Prieto D, Echavarria MP, Gallego JC (2021). Teleconsultation for outpatient care of patients during the Covid-19 pandemic at a University Hospital in Colombia. Int J Med Inform.

[25] Centro Nacional de Investigación en Evidencia y Tecnologías en Salud CINETS. Guías de Práctica Clínica para la Prevención, Detección Temprana y Tratamiento de las Complicaciones del Embarazo, Parto o Puerperio para uso de Profesionales de Salud. 2013. 84 p.

[26] Boelig RC, Saccone G, Bellussi F, Berghella V (2020). MFM guidance for COVID-19. Am J Obstet Gynecol MFM.

[27] Herrera JA, Gao E, Shahabuddin AKM, Lixia D, Wei Y, Faisal M (2006). Evaluación periódica del riesgo biopsicosocial prenatal en la predicción de las complicaciones maternas y perinatales en Asia 2002–2003. Colomb Med.

[28] World Health Organization. WHO Recommendations on Antenatal Care for a Positive Pregnancy Experience: Summary. Geneva, Switzerland: WHO 2018. Highlights and Key Messages from the World Health Organization’s 2016 Global Recommendations for Routine Antenatal Care. 2018. Available from: https://apps.who.int/iris/bitstream/handle/10665/259947/WHO-RHR-18.02-eng.pdf. [Cited 27 Jul 2021].

[29] Goyal M, Singh P, Singh K, Shekhar S, Agrawal N, Misra S (2021). The effect of the COVID-19 pandemic on maternal health due to delay in seeking health care: Experience from a tertiary center. Int J Gynecol Obstet.

[30] Coronavirus ( COVID-19 ) Infection in Pregnancy. R Coll Obstet Gynaecol. 2021;(February):1–98. Available from: https://www.rcog.org.uk/globalassets/documents/guidelines/2021-02-19-coronavirus-covid-19-infection-in-pregnancy-v13.pdf

[31] World Health Organization (WHO) (2020). Maintaining essential health services: operational guidance for the COVID-19 context. World Heal Organ.

[32] Montagnoli C, Zanconato G, Ruggeri S, Cinelli G, Eugenio A (2021). Restructuring maternal services during the covid-19 pandemic: Early results of a scoping review for non-infected women. Midwifery.

[33] Aziz A, Zork N, Aubey JJ, Baptiste CD, D’alton ME, Emeruwa UN (2020). Telehealth for high-risk pregnancies in the setting of the COVID-19 pandemic. Am J Perinatol.

[34] Leighton C, Conroy M, Bilderback A, Kalocay W, Henderson JK, Simhan HN (2019). Implementation and impact of a maternal-fetal medicine telemedicine program. Am J Perinatol.

[35] DANE. DIRECCIÓN DE CENSOS Y DEMOGRAFÍA ESTADÍSTICAS VITALES - EEVV [Internet]. Vol. 2019, CIFRAS DEFINITIVAS AÑO 2019. 2020. Available from: https://www.dane.gov.co/files/investigaciones/poblacion/cifras-definitivas-2019.pdf

[36] Campbell OMR, Cegolon L, Macleod D, Benova L (2016). Length of stay after childbirth in 92 countries and associated factors in 30 low- and middle-income countries: compilation of reported data and a cross-sectional analysis from nationally representative surveys. PLoS Med.

[37] Departamento Administrativo Nacional de Estadística. Boletín Técnico Estadísticas Vitales (EEVV). Bogotá D.C; 2022 Feb.

[38] Ministerio de Tecnologías de La Información y Comunicaciones. ¿Cómo está el país en conexiones de internet?. 2020. Available from: https://www.mintic.gov.co/portal/inicio/Sala-de-prensa/MinTIC-en-los-medios/151654:Como-esta-el-pais-en-conexiones-de-internet. [Cited 27 Jul 2021].

